# Reclaiming obsessionality: Beyond the obsessive‐compulsive spectrum

**DOI:** 10.1002/pcn5.70394

**Published:** 2026-08-19

**Authors:** Toshiyuki Kobayashi

**Affiliations:** ^1^ Department of Psychiatry Jichi Medical University Tochigi Japan

**Keywords:** middle voice, obsessionality, obsessive‐compulsive spectrum, psychopathology, repetitive behavior

## Abstract

The concept of the obsessive‐compulsive spectrum has exerted a major influence on contemporary psychiatry and contributed substantially to the establishment of the DSM‐5 category of obsessive‐compulsive and related disorders. However, the notion of obsessionality employed within this framework does not necessarily correspond to the classical psychopathological concept developed in European descriptive psychopathology. This paper reexamines the concept of obsessionality through a psychopathological comparison with neighboring phenomena, including impulsivity, addiction, autochthonous thinking, stereotypy, tic disorders, and motoric forms of obsessive‐compulsive disorder (OCD). Classical descriptions by Kraepelin, Jaspers, Schneider, and others suggest that obsessionality is characterized not merely by repetitive thoughts or behaviors, but by a distinctive cognitive structure involving compelling force, accompanying anxiety, awareness of irrationality, and an ego‐dystonic yet self‐attributed experience. Many conditions currently incorporated into the obsessive‐compulsive spectrum, particularly motoric OCD and related repetitive behaviors, appear to differ fundamentally from classical obsessionality and may be more closely related to impulsive, addictive, neurological, or developmental phenomena. To accommodate these diverse conditions, this paper proposes the provisional category of “middle‐voiced repetitive phenomena,” encompassing repetitive thoughts and behaviors that are neither fully voluntary nor wholly passive. We argue that the expansion of the obsessive‐compulsive spectrum has obscured the distinctive cognitive features that historically defined obsessionality. Returning to the classical psychopathological definition may therefore be useful for both clinical practice and research.

## THE IMPACT OF THE OBSESSIVE‐COMPULSIVE SPECTRUM CONCEPT

Several major changes were introduced in the classification system of the *Diagnostic and Statistical Manual of Mental Disorders, Fifth Edition* (DSM‐5).^1^ Among them was the removal of obsessive‐compulsive disorder (OCD) from the category of anxiety disorders and the establishment of a new chapter entitled *obsessive‐compulsive and related disorders* (OCRD). Furthermore, when one sees that disorders such as body dysmorphic disorder, trichotillomania, hoarding disorder, and excoriation disorder are included among the “related disorders,” it becomes apparent that the notion of obsession and compulsion employed here differs from that familiar to older generations of Japanese psychiatrists educated within a tradition grounded in German psychopathology. Is it really appropriate to place trichotillomania—where individuals may pull out their hair without conscious awareness—in the same category as OCD, in which a person washes their hands repeatedly despite knowing that no contamination is present?

Although DSM‐5 largely retains the DSM‐IV^2^ description of obsessions and compulsions, two notable statements found in DSM‐IV have disappeared. The first is that the individual “recognizes that the obsessional thoughts, impulses, or images are a product of his or her own mind” and the second is that “at some point during the course of the disorder, the person has recognized that the obsessions or compulsions are excessive or unreasonable.” Instead, DSM‐5 introduces insight specifiers that establish a spectrum ranging from good or fair insight to absent insight/delusional beliefs. Consequently, a diagnosis of OCD can now be made even when the patient does not regard the obsession as unreasonable. (Strictly speaking, DSM‐IV also allowed such diagnoses through the specifier “With Poor Insight.”)

As a result, the distinctiveness of obsessionality appears to have been attenuated. In this paper, we employ the term “obsessionality” to refer to the classical psychopathological concept represented by the German term *Zwang*, which denotes a pathological mode of experience encompassing both obsessional thoughts and compulsive acts. DSM‐5 now finds it necessary to provide a lengthy list of alternative psychiatric disorders under the caveat that the symptoms should not be better explained by another mental disorder. One is almost tempted to wonder whether DSM is in the process of dismantling the very concept of obsessionality itself.

Since Freud classified obsessive phenomena within the category of neurosis, conditions characterized primarily by obsessive symptoms were traditionally referred to as obsessive neurosis. Beginning with DSM‐III,^3^ however, the concept of neurosis was abandoned, and the condition corresponding to obsessive neurosis came to be designated as OCD. Obsessive symptoms are not specific to OCD; they may also be observed in schizophrenia, depressive disorders, eating disorders, and a variety of other psychiatric conditions.

Since the 1990s, a growing body of research^4^, ^5^, ^6^, ^7^ has attempted to reconceptualize obsessive phenomena under the rubric of the obsessive‐compulsive spectrum, ultimately contributing to the classification adopted in DSM‐5.^8^ The term obsessive‐compulsive spectrum disorders (OCSD) was introduced as a research construct encompassing conditions presumed to share underlying neurobiological mechanisms, such as dysfunction of the brain reward and impulse‐control systems. Accordingly, the proposed spectrum extended beyond OCD to include disorders such as eating disorders, pathological gambling, and tic disorders.^4^ During the development of DSM‐5, there was initially a proposal to adopt OCSD as a separate diagnostic chapter.^9^ Ultimately, however, DSM‐5 adopted the more narrowly defined clinical category of OCRD instead, without committing to the specific neurobiological mechanisms postulated by the OCSD concept.^10^ These disorders are described as sharing not only obsessions and compulsions but also preoccupations and repetitive behaviors or mental acts associated with those preoccupations. They are further characterized by repetitive body‐focused behaviors, such as hair pulling or skin picking, together with repeated attempts to reduce or stop these behaviors. Thus, DSM‐5 effectively treats these related disorders as belonging to a broader domain centered on pathological preoccupation and repetitive behavior. Yet whether framed as OCSD or OCRD, the concept of obsession and compulsion invoked here appears to have moved away from the traditional concept of obsessionality developed in classical European, particularly German, psychopathology.

Is obsessionality fundamentally a neurotic phenomenon, or is it better understood as a neurological abnormality? When one encounters the intense restricted interests and repetitive concerns often observed in individuals with autism spectrum disorder, it becomes difficult to determine whether such preoccupations should be classified as obsessions—that is, interpreted in terms of neurotic mechanisms—or whether they represent a distinct type of neurological dysfunction. To be sure, neurosis and neurological impairment need not be regarded as mutually exclusive categories. Yet one is increasingly left wondering what exactly is being described by the terms “obsession” and “compulsion,” in the first place.

In this paper, we examine several concepts adjacent to obsessionality, explore how they differ from obsessive phenomena proper, reconsider what should legitimately be regarded as “obsession,” and finally present the author's own perspective on the matter.

## CLASSICAL DEFINITIONS OF OBSESSIONALITY

In clinical practice, psychiatrists have long described obsessive symptoms in individuals with autism spectrum disorder and obsessive traits in anorexia nervosa. Yet, while doing so, we have often employed the term *obsession* despite harboring at least some doubt as to whether these phenomena are truly identical to the symptoms observed in patients traditionally diagnosed with obsessive neurosis. Likewise, clinicians occasionally encounter patients whose repetitive behaviors, initially regarded as compulsions, upon closer examination appear more consistent with tics or impulsive acts. In other cases, it remains genuinely difficult to determine whether a patient's repetitive behavior should be understood as a compulsion or as an addiction. Such clinical observations suggest that the proponents of the OCSD concept are not without justification. Nevertheless, unless these diverse phenomena can be shown to share a common neurobiological substrate and to respond to the same therapeutic interventions, there is little clinical value in subsuming them under a single diagnostic construct. At present, careful phenomenological differentiation among these symptoms is likely to be of greater clinical importance than grouping them together.

It is therefore worthwhile to revisit the classical descriptions of obsessionality in German psychopathology.

Emil Kraepelin wrote of obsessive neurosis: “Under the designation of obsessive neurosis, we group together a number of morbid states whose common characteristic is a vivid sense of being overwhelmed by intrusive ideas or fears.”^11^ The emphasis here is placed on the overwhelming force of intrusive ideas and fears. The German term *Zwang*, conventionally rendered in English as *obsession* or *compulsion* depending on the context, derives from the verb *zwingen* (“to force” or “to compel”). In Japanese, the term corresponding to the German *Zwang* is *kyōhaku* (強迫). As in German, it serves as a superordinate concept encompassing both obsessions and compulsions. The Japanese term *kyōhaku*, inherited from classical Chinese, originally denotes being compelled or forced against one's own will. Likewise, the English terms *obsession* and *compulsion* retain connotations of being beset by something and being compelled, respectively.

Karl Jaspers described obsessive phenomena as follows: “The patient believes contents that are often of considerable significance and yet simultaneously knows that they are false”; furthermore, “the patient's thoughts are gathered around a single fundamental idea that continually returns to consciousness against the will (*thought compulsion*), and although the patient is convinced that the idea is incorrect, it nevertheless forces itself upon him as though it were true (*compulsion of validity*).”^12^

The crucial point here is that the patient both believes and disbelieves the idea at the same time. What lends the idea its apparent credibility is precisely the force with which it presses itself upon consciousness.

Kurt Schneider, arguing that only the core of obsession can be defined, wrote: “An obsession is the inability to free oneself from a content of consciousness despite judging it to be meaningless in itself, or at least dominant and persistent without adequate reason.”^13^

The central issue emerging from these descriptions is relatively simple. Obsessions are recurrent ideas that impose themselves upon the individual with compelling force, while at the same time being recognized as erroneous. To this one might add the anxiety that accompanies them or forms their background, together with their characteristic quality of being ego‐dystonic yet experienced as belonging to oneself.

Such a characterization of obsessionality was long regarded as common knowledge within Japanese psychiatry. Evidence of this can be found in the entry for “obsession” in the Japanese *Dictionary of Contemporary Psychiatry*, which states: “Thoughts, mental images, or impulses that arise repeatedly and persistently. The inability to refrain from thinking them is termed obsessive thinking…. Although patients usually recognize such thoughts as irrational and absurd, they are unable to exclude them from consciousness. Obsessions are ego‐dystonic and unacceptable to the patient, accompanied by anxiety, discomfort, or fear; nevertheless, they are experienced as belonging to the self.”^14^

Nor is this understanding unique to Japanese psychiatry. Campbell's English‐language psychiatric dictionary defines obsessions as “thoughts, feelings, or impulses that repeatedly and persistently force their way into consciousness, even though they are unwelcome.”^15^ Similarly, Porot's French psychiatric dictionary describes them as “an idea, or a series of ideas, that persistently troubles and distresses the mind and that the subject is unable to dismiss despite recognizing it as absurd.”^16^ Peters' German psychiatric dictionary, under the entry *Zwangsphänomene* (“obsessive phenomena”), defines them as “a collective term for representations and impulses to action that impose themselves upon a person and whose occurrence cannot be successfully resisted.”^17^

The elements common to these definitions may be summarized as follows:

(1) Repetitiveness.
(2) Compelling force.
(3) Accompanying anxiety.
(4) Awareness of irrationality.
(5) Ego‐dystonicity combined with a sense of ownership.

These characteristics are not independent of one another. The awareness of irrationality may arise precisely because the experience is felt as compelling, while the sense of ego‐dystonicity may in turn stem from the recognition of its irrationality.

In the classical psychopathological texts discussed above, although the term *obsession* or *compulsion* is used broadly, the focus is directed primarily toward obsessive ideas, as if compulsive acts were regarded as secondary phenomena. In Japanese and German, where a single term—*kyōhaku* or *Zwang*—can refer to both obsessions and compulsions, the distinction is less explicit. By contrast, in English and French, where separate terms exist for these phenomena, the very structure of the language seems to imply a conceptual hierarchy in which obsessions are primary and compulsions arise secondarily. In other words, an obsession that haunts or possesses the individual compels a compulsive act. Yet even in Japanese and German psychiatry, ideas and actions do not appear to occupy equal positions under the rubric of *kyōhaku* or *Zwang*. Thus, the *Dictionary of Contemporary Psychiatry* defines a compulsive act as “a disturbance of behavior occurring in association with an obsession,”^14^ thereby explicitly assigning primacy to the obsession.

There is no difficulty in acknowledging the existence of obsessions that do not lead to compulsive acts. One may, for example, encounter a patient who is preoccupied with contamination yet does not engage in compulsive washing. But can there be compulsive acts in the absence of obsessions? Suppose a person washes their hands repeatedly but explains only that, for some unknown reason, they cannot refrain from doing so. Would we call this a compulsive act, or would we instead describe it as a stereotyped behavior or an impulsive act? In some cases, what Lacan referred to as *passage à l'acte* ^18^ may represent yet another possibility. Indeed, the psychopathological nature of such acts may be considerably more fluid than our diagnostic terminology would suggest.

## IMPULSES, ADDICTIONS, AUTOCHTHONY, AND STEREOTYPY

These clinical observations suggest that, although compulsive acts and impulsive acts are conceptually opposed, they may in fact form a clinical spectrum. The contrast between *compulsivity* and *impulsivity* is particularly appealing in this regard. As Siddiqui et al. have noted: “The relationship between compulsive and impulsive disorders is more complex. Some authors have suggested that compulsivity and impulsivity are orthogonal dimensions and that patients can have both compulsive and impulsive symptoms. Others have argued that compulsive and impulsive disorders lie at opposite ends of a unidimensional spectrum.”^19^

According to the latter view, impulsivity and compulsivity may be conceptualized as opposite poles of a spectrum, with impulsivity characterized by an underestimation of harm and risk, and compulsivity by an overestimation of harm and excessive avoidance of risk.^20^ Put differently, impulsivity involves doing what one ought not to do, whereas compulsivity involves being unable to do what one wishes to do because of fear. In the former, desire or drive is prominent; in the latter, anxiety occupies a central position.

Hollander and Benzaquen, who played a major role in the development of the concept of OCSD, located the core of the spectrum in an impaired ability to delay or inhibit repetitive behaviors, including compulsive acts, impulsive acts, and tics.^21^ Yet such a definition risks becoming indistinguishable from mere repetitive behavior. Consequently, it becomes necessary to take into account the anxiety or distress generated by obsessions.^22^ This leads to the formulation discussed earlier in terms of “preoccupation” and “repetitive behavior.” From this perspective emerges the distinction between, on the one hand, the prototypical cognitive form of OCD, characterized by obsessions and cognitive processes that amplify anxiety, and, on the other, a motoric form of OCD characterized by sensory phenomena such as the pursuit of a “just‐right feeling” and the relief of feelings of incompleteness, generally accompanied by ego‐syntonicity and poor insight.

However, as Starcevic and Janca have pointed out, many obsession‐like behaviors observed in OCSD depart from the classical concept of obsession in that they provide pleasure or satisfy an urge, impulse, or craving.^23^ Such phenomena cannot easily be understood as arising from the anxiety that traditionally forms the background of obsessive symptoms. In particular, motoric OCD comes remarkably close to what might be called “a compulsion without an obsession,” raising the question of whether such behavior ought instead to be regarded as impulsive.

Let us therefore examine the concept of impulse in greater detail. As noted above, the term *impulse* is itself included in classical definitions of obsession. According to the *Dictionary of Contemporary Psychiatry*, the category of “obsession” encompasses thoughts, mental images, and impulses; DSM‐IV employs a similar formulation. The term “impulse” also appears in the definitions of obsession provided by Campbell and Peters.

Interestingly, the *Dictionary of Contemporary Psychiatry* contains no independent entry for “impulse” itself, but only for “impulsive act.” There, an impulse is described as a psychoanalytic term borrowed from the notion of an impulse traveling along a nerve fiber and refers to “a movement of the mind that seems compelled to discharge itself in action.”^14^ Because impulses originate in the id, they are often treated as synonymous with wishes, desires, or drives. When they pass into action without undergoing sufficient scrutiny by the ego, the resulting behavior is termed an impulsive act.^14^

It is precisely because an impulse is conceived as a mental movement directly and compulsively linked to action that the notion of impulse is incorporated into definitions of obsession, particularly those forms of obsession that culminate in compulsive acts. The crucial distinction between impulse and obsession, however, is that although both are experienced as belonging to oneself, obsessions are ego‐dystonic whereas impulses are ego‐syntonic. Indeed, one might go further: impulses proceed directly to action without even becoming the object of reflection as to whether they are ego‐syntonic or ego‐dystonic.

At its core, an impulse involves carrying out an action that the subject desires. In the absence of any prohibition or restraint, it would be little different from an ordinary wish or desire. Such restraints may be moral, social, economic, or of many other kinds. What characterizes an impulse is that the action is carried out despite these restraints. In this sense, the impulse itself does not inherently entail conflict. If conflict arises, it does so either when the individual attempts to resist acting upon the impulse or after having surrendered to it. Accordingly, there is ordinarily no such thing as an “impulsive idea”; once an impulse emerges, it tends to proceed directly into action, forming an inseparable unity with the impulsive act itself.

One can, of course, imagine a state in which an individual remains in conflict while struggling to prevent an impulse from being translated into action. Such a condition has been described as an *obsessive impulse* or *obsessive drive*. However, insofar as it consists of “an impulse to perform a particular act recurring repeatedly against one's will,”^14^ it may reasonably be regarded as falling within the domain of obsessionality.

Although actual usage of these terms is often inconsistent, the conceptual distinction between obsessionality and impulsivity may be summarized as follows. Obsessionality is ego‐dystonic, whereas impulsivity is ego‐syntonic. Obsessional phenomena give rise to substantial conflict under the scrutiny of the ego, whereas impulsive phenomena tend to pass directly into action with relatively little intervention by such self‐reflective processes.

The spectrum between obsessionality and impulsivity discussed above may therefore be understood as a continuum ranging from ego‐dystonicity to ego‐syntonicity, or alternatively as a continuum defined by the degree to which mental contents are subjected to scrutiny by the ego.

Addiction may also be included among the phenomena in which a person engages in an act despite the presence of prohibitions or restraints. The *Dictionary of Contemporary Psychiatry* notes that the word *addiction* is derived from a Latin word meaning “to be given over” or “to be devoted.” It further observes that the term has come to acquire a meaning overlapping with that of obsessionality, namely, “being possessed by something from which one cannot detach oneself.”^14^ At the same time, addiction is distinguished from obsessional behavior in that it is something into which the individual willingly immerses himself or herself—that is, it is ego‐syntonic—whereas obsessional behavior is experienced as aversive and ego‐dystonic despite its repetitive nature.^14^ Addiction is further described as arising from a disturbance of impulse control.^14^

While the term *impulse* may refer to a single, sudden event, addiction may be understood as a chronic condition consisting of a continuous succession of impulses. Nevertheless, the boundary between addiction and impulsivity remains indistinct.

The boundary between addiction and obsessionality may likewise become blurred. As suggested by the definition cited above, one might provisionally distinguish them by saying that drinking alcohol is undertaken because it is experienced as desirable, whereas compulsive washing is performed because the individual feels compelled to do something undesirable. On closer examination, however, the distinction is far from straightforward. People drink because they want to drink, yet often do so while aware of prohibitions and restraints—for example, that drinking is harmful to their health or interferes with their work. Indeed, one form of obsessionality may consist precisely in a desire to transgress a prohibition, a tendency that may become stronger the more powerful the prohibition itself. To the extent that this aspect predominates, addiction itself may come to appear obsessional.

Conversely, compulsive washing may, over the course of chronic illness, acquire a rather different quality. Even while the skin of the hands becomes severely damaged, the individual may become deeply absorbed in the act of washing. To say that the person engages in it joyfully would perhaps be an exaggeration; nevertheless, immersion in washing may become so complete that concerns about contamination recede into the background. Such absorption in handwashing begins to resemble, in certain respects, the state of being immersed in alcohol.

A further concept must also be taken into consideration: **autochthonous phenomena**. If one accepts that there are *autochthonous thoughts* but not, in ordinary usage, *autochthonous acts*, then autochthonous thought may be viewed as a candidate for filling the conceptual vacancy of a “pure impulse that does not culminate in action.” However, according to the *Dictionary of Contemporary Psychiatry*, autochthonous thought is defined as “an experience in which thoughts arise naturally one after another,” involving “a continuous and automatic emergence of various disconnected thoughts to which the individual pays no particular attention and perceives no meaningful relation.”^14^ Its characteristic feature is therefore the relative absence of the compelling force that characterizes obsessions and impulses.

The concept of autochthonous thought originated with Carl Wernicke,^17^ and appears to have been used primarily in German and Japanese psychopathology. In Campbell's psychiatric dictionary, the term *autochthonous* is employed only in the sense of “primary,” and not as the designation of a specific psychopathological phenomenon.^15^ In the French‐speaking tradition, the phenomenon corresponding most closely to autochthonous thought would probably be *mentisme*,^24^ yet the notion of “autochthony” itself does not appear as an independent concept.

It was Nobuo Nakayasu who extended the notion of autochthony beyond the domain of thought.^25^ Nakayasu pointed out that many phenomena described in earlier literature as obsessive symptoms in schizophrenia are in fact better understood as autochthonous experiences, and argued that obsessionality and autochthony constitute distinct symptom series.^26^ In his account of obsessionality, Nakayasu emphasized the presence of the experience of being compelled, driven, or pressed by something. Among the five characteristics of classical obsessionality identified above, this corresponds to the second feature, namely compelling force, which he regarded as a necessary condition of obsessionality. This would seem to be a reasonable position insofar as it provides a clear basis for distinguishing obsessionality from autochthony.

A further concept that must be considered is **stereotypy**. According to the *Dictionary of Contemporary Psychiatry*, stereotypy is “a phenomenon in which behavior, posture, or speech is repeated in an identical and unchanging manner,” characterized by the fact that it is “without purpose or utility and is not adapted to the surrounding circumstances.”^14^

The term *stereotypy* is generally applied when it is unclear what purpose a particular behavior, posture, or utterance serves and when no subjective account can be obtained from the individual concerned. If the person's inner experience were accessible, the phenomenon might instead be described using another term, such as impulse or addiction. Thus, stereotypy may in some cases represent less a distinct experiential category than a descriptive label applied in the absence of information concerning subjective motivation.

Stereotypies are observed in severe or advanced schizophrenia, severe intellectual disability, encephalitis, Parkinson's disease, and frontotemporal dementia, among other conditions.^14^ The term is therefore most often employed when the phenomenon is thought to arise primarily from neurological dysfunction rather than from psychological processes in the narrower sense.

In light of the foregoing discussion, the concept of the obsessive‐compulsive spectrum warrants reconsideration. It seems problematic to subsume **motoric OCD** within the concept of obsessionality. In DSM‐5, the contents of OCD include contamination, forbidden or taboo thoughts, and fears of causing harm, as well as symmetry‐related symptoms, consisting of obsessions concerning symmetry and compulsions such as repeating, arranging, and counting.^1^ Thus, phenomena corresponding to motoric OCD appear to be incorporated within the category of OCD itself. However, the pursuit of a *just‐right feeling* is motivated by the satisfaction derived from attaining that feeling; it is not necessarily driven by anxiety about things being “not just right.” To the extent that motoric OCD is characterized by sensory phenomena such as the pursuit of a *just‐right feeling* or the relief of incompleteness, and by relatively ego‐syntonic features and poor insight,^26^ it appears closer to impulsivity or addiction than to classical obsessionality. Cases certainly exist in which the absence of a *just‐right feeling* gives rise to anxiety, but should these not be regarded as symptoms of a different nature? In the classical textbooks,^12^, ^13^ symptoms corresponding to motoric OCD receive little attention; at most, Kraepelin mentioned “arithmomania” or obsessive counting.^11^ At the very least, motoric OCD and symmetry‐related symptoms appear to require further conceptual clarification.

Similarly, among the disorders classified in DSM‐5 as OCRD, trichotillomania, hoarding disorder, and excoriation disorder seem more closely related to disturbances of impulse control or to addictive phenomena. Aoki et al.,^27^ discussing obsession‐like symptoms in developmental disorders, argued that whereas obsessive symptoms are ego‐dystonic, *kodawari* (“fixation” or “preoccupation”) is ego‐syntonic, and suggested that there may exist a group of conditions better understood as a **“kodawari spectrum”** rather than an obsessive‐compulsive spectrum. Put differently, this is a contrast between *toraware* (“being seized by” or “being trapped in”) and *kodawari* (“fixation” or “preoccupation”). From the perspective developed above, such a distinction might appear reducible to that between obsessionality and impulsivity/addiction. However, Aoki et al.'s concept of *kodawari* is notable for the near absence of any element of prohibition or restraint. If impulsivity and addiction are characterized by actions that break through prohibitions, then *kodawari* may in fact be closer to **autochthony** than to impulsivity.

As noted above, Hollander and colleagues, who proposed the concept of the obsessive‐compulsive spectrum, located its essential feature in a diminished capacity to delay or inhibit repetitive behaviors. If this definition is accepted, however, the spectrum inevitably comes to include a wide variety of states described by such terms as impulsivity, addiction, autochthony, and stereotypy. In effect, the spectrum arranges diverse symptoms according to varying degrees of obsessionality, yet in doing so places at its periphery phenomena that can scarcely be regarded as obsessional. What these phenomena actually share is not obsessionality itself, but rather the repetitive emergence of thoughts or actions. Moreover, these thoughts and actions possess a mode of occurrence that distinguishes them from intentional, volitional acts.

With regard to addiction, one is inclined to assume that drinking is a voluntary and intentional act. Yet, as Koichiro Kokubun has argued, when one examines actual clinical cases, it is difficult to maintain that the individual is simply drinking of his or her own free will; indeed, such a view leaves little room for therapeutic intervention.^28^ According to Kokubun, the state in which the drinker finds himself or herself is neither active nor passive, but rather one that may be described in terms of the **middle voice**. The same may be said of the obsessive‐compulsive spectrum discussed in this paper, as well as of the related phenomena of impulsivity, addiction, autochthony, and stereotypy. If these concepts are to be understood collectively, their common feature is that they cannot be regarded as fully voluntary thoughts or actions, yet neither are they passive experiences in the sense of experiences of external control. The term **middle‐voiced** aptly captures this mode of experience.

Furthermore, these phenomena come to be described as pathological not because they occur only once or on rare occasions, but because they recur repeatedly. If so, it may be reasonable, as a provisional designation, to group them together under the heading of **middle‐voiced repetitive phenomena**.

It should be emphasized, however, that this article does not propose a new spectrum of disorders. Rather, these phenomena, which share certain overlapping features, are provisionally brought together on the basis of their phenomenological similarities—that is, similarities evident in clinical observation. The subsequent task is to differentiate them once again, with particular attention to obsessionality, thereby enabling a more refined psychopathological diagnosis.

## MIDDLE‐VOICED REPETITIVE PHENOMENA AND OBSESSIONALITY

In *Philosophical Investigations*, Ludwig Wittgenstein writes: “But let us not forget this: when ‘I raise my arm,’ my arm goes up. And the problem arises: what is left over if I subtract the fact that my arm goes up from the fact that I raise my arm?”^29^ In Wittgenstein's discussion, what remains would presumably be nothing more than a difference in language‐games and grammatical constructions. From a commonsense perspective, however, what remains is the will. Yet when the arm is raised as a compulsive act, what remains can scarcely be described as will. According to Koichiro Kokubun, most modern languages contrast the active and passive voices, thereby bringing the problem of will into sharp focus. In Ancient Greek, however, and in even older languages, the contrast was between the active and the middle voices. Within the opposition between active and middle voices, the notion of will does not emerge as a distinct entity; indeed, Kokubun notes that Ancient Greek possessed no word corresponding to the modern concept of will.

Kokubun^28^ argues that impersonal verbs, intransitive verbs, and passive constructions developed out of the middle voice, and further suggests the possibility that even the active voice itself may have arisen from it. In the present discussion, however, I wish to focus less on the meanings derived from the middle voice—such as spontaneity, possibility, or intransitivity—than on its developmental stage of emergence. If we trace language back to its origins, it is plausible to imagine that language began when an observed action—for example, running—was simply designated as “running.” At that stage, the verb “to run” would have had no subject, and consequently neither active nor passive voice. Such a usage would be middle‐voiced, and the notion of free will would not yet have emerged.

A similar consideration may apply to our individual experience. Acts that are subsequently designated as “running” may first arise without a subject—in other words, without will. This corresponds to the view that human action is fundamentally autochthonous rather than volitional.^28^ For the purposes of the present discussion, thought may likewise be treated as a form of action, namely the act of thinking. People believe that they act through free will, yet in everyday life they are often compelled to perform actions they would rather avoid, find themselves carrying out actions different from those they intended, or behave “unconsciously.” Nevertheless, after the fact they are able to claim that they acted of their own will. One might also recall the famous experiment of Benjamin Libet, which demonstrated that brain activity associated with an action begins approximately 350 ms before the subject becomes aware of the intention to act.^30^ In other words, when an action occurs, the determination of whether it was a voluntary and intentional act, something done impulsively, something done under irresistible pressure, or something imposed by another person—that is, the attribution of agency and the localization of the process leading to the act—is a cognitive operation performed retrospectively^31^.

Taken to its extreme, however, such a position would depart too far from ordinary experience. There are certainly occasions on which one forms an intention, acts upon it, and successfully carries out the intended action. The cognitive systems responsible for monitoring and regulating action are therefore not entirely uninvolved in behavior. Rather, they likely participate in processes such as the planning of a sequence of actions or the generation of subsequent actions following an initial one. The relationship is not linear but resembles what might be described as a state of mutual interpenetration. Nevertheless, many everyday actions arise without clearly formulated intentions, and intentions are subsequently constructed after the fact.

Although it is beyond the scope of the present paper to clarify further the nature of action in normal experience, the situation appears different in the case of **middle‐voiced repetitive phenomena**. Here, actions arise without a clearly articulated intention, and, moreover, they are not accompanied by the cognitive labeling that identifies them as active, voluntary acts. The actions therefore retain their middle‐voiced character. Whether one is dealing with obsessionality or impulsivity, as long as one recognizes only the dichotomy between activity and passivity, such phenomena must be regarded as one's own actions. Yet it is equally clear that they cannot be considered ordinary voluntary and intentional acts. The concept of the **middle voice** introduces a third term into the opposition between activity and passivity, thereby providing a means of describing states that belong fully to neither category.

For the sake of conceptual clarity, it is useful to distinguish between two separate issues: first, the repeated occurrence of actions—including what may be called “acts of thinking”—and second, the way in which the subject cognitively apprehends those actions. In stereotypies and repetitive behaviors driven by a *just‐right feeling*, introspection regarding the action itself is generally limited. In contrast, in classical obsessionality, its defining characteristics can scarcely be described except at the level of cognition. Obsessionality may therefore be defined by features (2)–(5): Compelling force, Accompanying anxiety, Awareness of irrationality, and Ego‐dystonicity combined with a sense of ownership.

In obsessional thoughts, a particular thought presses itself upon the subject with such force that, despite being recognized as irrational, it is difficult to experience it as something actively and voluntarily thought by the ego. This phenomenon may be interpreted from a linguistic perspective. Consider, for example, the thought, “My hands are dirty.” For this thought to constitute an obsession, it is essential that it be accompanied by awareness of its irrationality: the subject knows that “my hands are not dirty,” yet cannot dismiss the intrusive thought. Rather than assuming that a separate thought such as “That is absurd” simultaneously arises as an awareness of irrationality, it may be more accurate to suppose that the negation of the original thought—“my hands are not dirty”—arises simultaneously with it. In such a state, affirmation and negation coexist. Their logical antagonism may give rise to the peculiar experience in which a thought presses itself upon consciousness with irresistible force while at the same time being recognized as irrational.

Actions themselves cannot, of course, possess affirmative and negative forms in the same way that thoughts do. Nevertheless, behind a compulsive act one may discern a conflict between the affirmative and negative forms of a volitional thought—for example, “I should wash” and “I should not wash.” Without such an antagonism in the background—in other words, without an obsessional thought—the behavior would not be a compulsive act but rather some other form of middle‐voiced repetitive phenomenon, to be described according to its particular characteristics as an impulsive act, a stereotyped act, or something similar.

Feature (1), repetitiveness, is common to all middle‐voiced repetitive phenomena. It may be assumed that difficulties arise in the systems responsible for monitoring actions, correcting them, and inhibiting subsequent actions. However, it is unlikely that the nature of this disturbance in control is identical across all of the conditions grouped here under the heading of middle‐voiced repetitive phenomena. For this reason, it would be an oversimplification to gather them together under a single label such as the *obsessive‐compulsive spectrum*.

In obsessionality, the compelling nature of the experience, the accompanying anxiety, and the awareness of its irrationality are understood to generate conflict, which in turn gives rise to the repetition of thoughts and actions. This is fundamentally a neurotic model and is clearly inapplicable to fixations based on a *just‐right feeling*. Given that behavior patterns rooted in such fixations—namely, what has been termed motoric OCD—are frequently observed in neurological conditions such as tic disorders and in neurodevelopmental disorders such as autism spectrum disorder, it is difficult to avoid the conclusion that the repetitive behaviors seen in these conditions arise from some form of dysfunction within neural systems.

At the same time, in conditions such as high‐functioning autism spectrum disorder, psychologically mediated repetitive symptoms and neurologically based repetitive symptoms may coexist in ways that are difficult to disentangle. For this reason, the mere presence of a developmental disorder does not justify the conclusion that repetitive phenomena observed in such patients cannot represent classical obsessionality. It is precisely this overlap that complicates clinical practice.

Furthermore, reports continue to appear describing symptoms that closely resemble classical obsessionality in conditions for which biological mechanisms are presumed.^32^, ^33^, ^34^, ^35^ In addition, not only can classical obsessionality respond to pharmacotherapy, but neuromodulatory interventions have also been reported to be effective in some cases.^32^, ^36^, ^37^ Such findings suggest that classical obsessionality itself may possess a neurological substrate. Conversely, reports of cases in which psychological factors—including bodily conditions—appear to contribute to the onset^38^ or remission^39^ of symptoms indicate a substantial role for psychological mechanisms. Classical obsessionality may itself arise from varying combinations of psychological and neurological mechanisms.

The argument of the present paper, however, is that this does not justify the attempt, embodied in the concept of the obsessive‐compulsive spectrum, to subsume all repetitive phenomena under a single framework based merely on repetition. Such an approach seems premature and of limited value. When symptoms belonging to the category of middle‐voiced repetitive phenomena proposed in this paper are encountered, it may be clinically and scientifically fruitful to return to the classical definition of obsessionality and determine whether they represent obsessionality proper or another repetitive behavioral syndrome.

## CONCLUSION

In summary, obsessionality refers to those conditions within the broader category of middle‐voiced repetitive phenomena—a category that encompasses states ranging from psychological mechanisms to neurological abnormalities and is characterized by the repetitive occurrence of thoughts and actions—that possess a distinctive cognitive pathology. It is precisely this cognitive dimension that has traditionally constituted the defining core of obsessionality. By contrast, when one attempts, as in the concept of the obsessive‐compulsive spectrum, to subsume these phenomena within a single overarching framework, the cognitive elements that form the core features of obsessionality are reduced to merely one aspect among many, while emphasis shifts toward the repetitive behaviors themselves. As a consequence, the essential nature of obsessionality risks being obscured.

## AUTHOR CONTRIBUTIONS

N/A.

## CONFLICT OF INTEREST STATEMENT

The author declares no conflicts of interest.

## ETHICS APPROVAL STATEMENT

N/A.

## PATIENT CONSENT STATEMENT

N/A.

## CLINICAL TRIAL REGISTRATION

N/A.

## Data Availability

Data sharing not applicable to this article as no datasets were generated or analyzed during the current study.
